# Supplementary material for: Structural and molecular basis for Cardiovirus 2A protein as a viral gene expression switch
Source: Nat Commun. 2021 Dec 9;12:7166. doi: 10.1038/s41467-021-27400-7 (PMC8660796; doi:10.1038/s41467-021-27400-7)
Supplement: Supplementary file 1 — Supplementary Information [file 41467_2021_27400_MOESM1_ESM.pdf]

Supplementary Figures

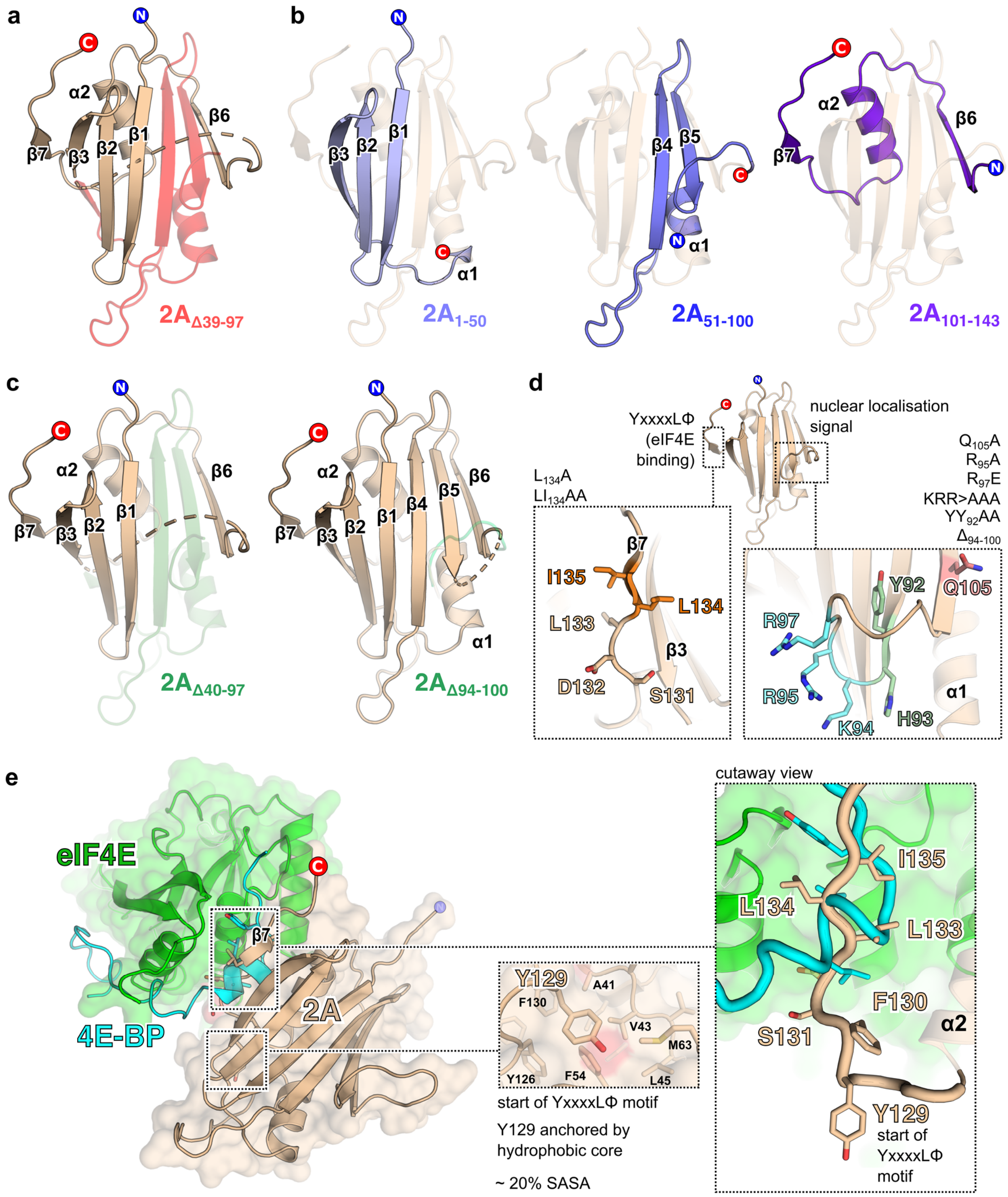

Supplementary Figure 1 – related to **Figure 1. Details of previously described 2A sequence motifs, deletions and point mutations. a,** Structural consequences of the $2A_{\Delta 39-97}$ mutation described by Svitkin *et al* [1]. Deleted amino acids are highlighted in red. **b,** Truncation fragments $2A_{1-50}$, $2A_{51-100}$ and $2A_{101-143}$ described by Petty *et al* [2]. In each case the remaining fragment is highlighted in blue and overlaid against the structure of the full protein for context. **c,** Deletion mutants $2A_{\Delta 40-97}$ and $2A_{\Delta 94-100}$ as described by Groppo *et al* [3]. Deleted

amino acids are highlighted in green. **d,** Location of point-mutations made by Groppo *et al* in the putative nuclear localisation sequence and putative C-terminal YxxxxLΦ eIF4E binding motif. Mutated amino acids are shown as coloured sticks. **e,** Comparison of 4E-BP1 YxxxxLΦ binding motif and the putative YxxxxLΦ motif in 2A. The crystal structure of the complex between eIF4E (green) and 4E-BP1 (blue) is shown (Siddiqui et al., 3U7X) with 2A (wheat) docked via least-squares superposition of the YxxxxLΦ motif. <*Insets*> Contrast between the 2A YxxxxLΦ motif, in an extended β-strand conformation (wheat), and the 4E-BP1 YxxxxLΦ motif, in a compact α-helical conformation, with Y129 partially buried (~ 20% solvent-accessible surface area; SASA). 2A binding to eIF4E is thus not compatible with the known 4E-BP1 interface without substantial conformational rearrangement.

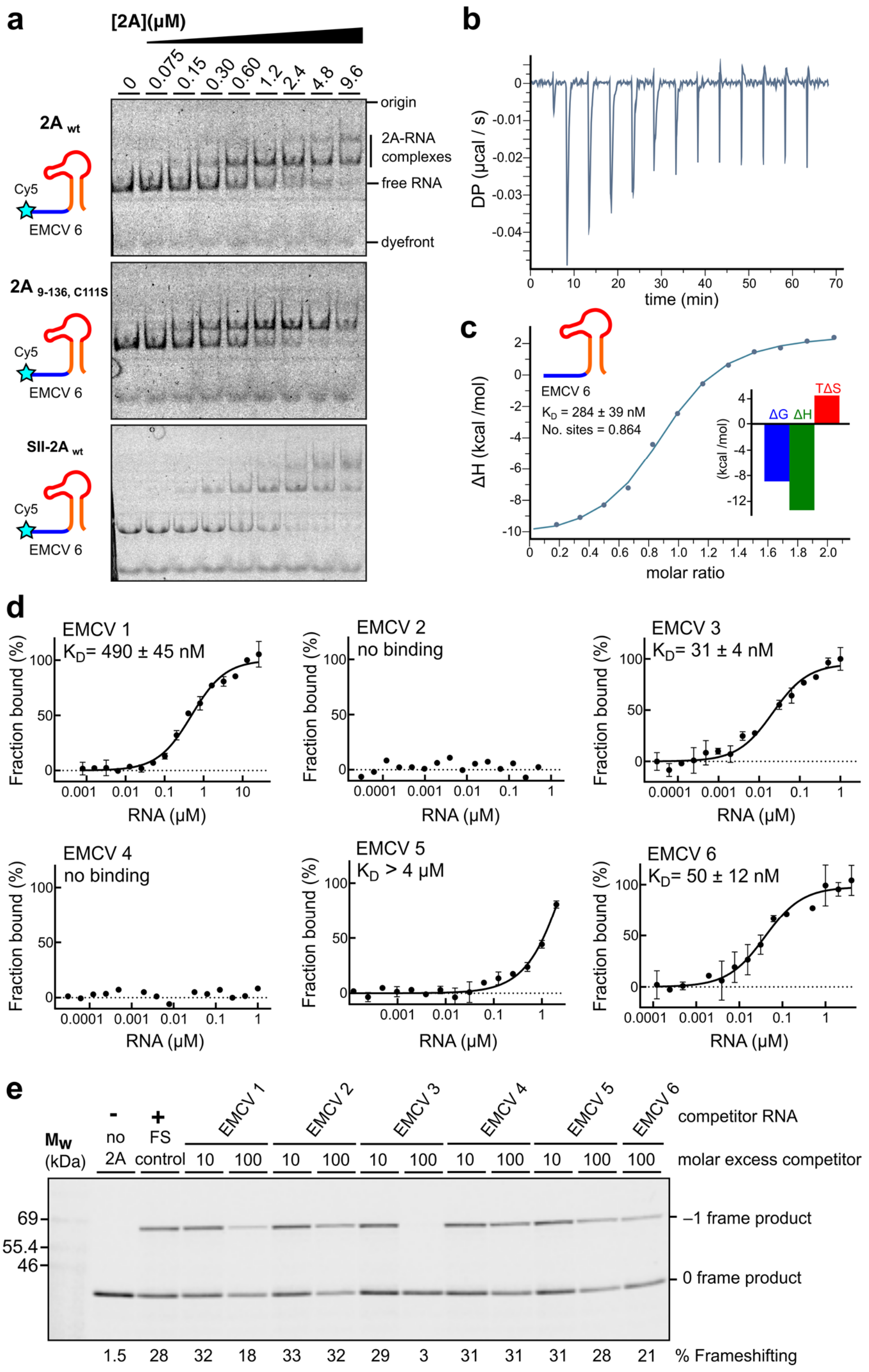

Supplementary Figure 2 – related to **Figure 2. The 2A-RNA interaction does not require disulfide bond formation and is an exergonic process with nanomolar affinity and 1:1 stoichiometry. a,** Side-by-side comparisons of $2A_{wt}$, $2A_{9\text{-}136;\ C111S}$ and $SII\text{-}2A_{wt}$. Equivalent RNA binding is observed in all cases by EMSA analyses conducted with 50 nM Cy5-labelled EMCV 6 RNA and 2A concentrations between zero and 9.6 μM. Following non-denaturing electrophoresis, fluorescence was imaged using a Typhoon scanner. Showing representative gels from two independent experiments. **b,** Baseline-corrected differential power (DP) versus time for ITC titration of EMCV 6 RNA into 2A protein. **c,** Normalized binding curve showing integrated changes in enthalpy (ΔH) against molar ratio for titration in **b**, showing a ~1:1 molar

ratio and nanomolar affinity <*Inset*> Histogram showing relative contributions of ΔH and TΔS terms to the overall exergonic interaction. **d,** MST binding curves and reported K_D values of fluorescently labelled 2A protein (5 nM) and short unlabelled RNAs (as in **Fig. 2a, b**) at concentrations between 800 pM – 26 µM for EMCV 1 and 120 pM – 4 µM for EMCV 2–6. All measurements were repeated as two independent experiments and error bars represent the standard deviation from the mean. **e,** Experiment showing the effects of titrating excess short RNAs (EMCV 1–6) as competitors into an *in vitro* frameshift reporter assay. The concentrations of the reporter mRNA and 2A were kept constant in the RRL and short RNAs were added in 10- and 100- fold molar excess relative to the reporter mRNA, as indicated. Translation products were visualised by using ^{35}S-Met autoradiography, and % frameshifting was calculated following densitometry and correction for the number of methionines present in 0 frame and –1 frame products. Showing representative gel from two independent experiments.

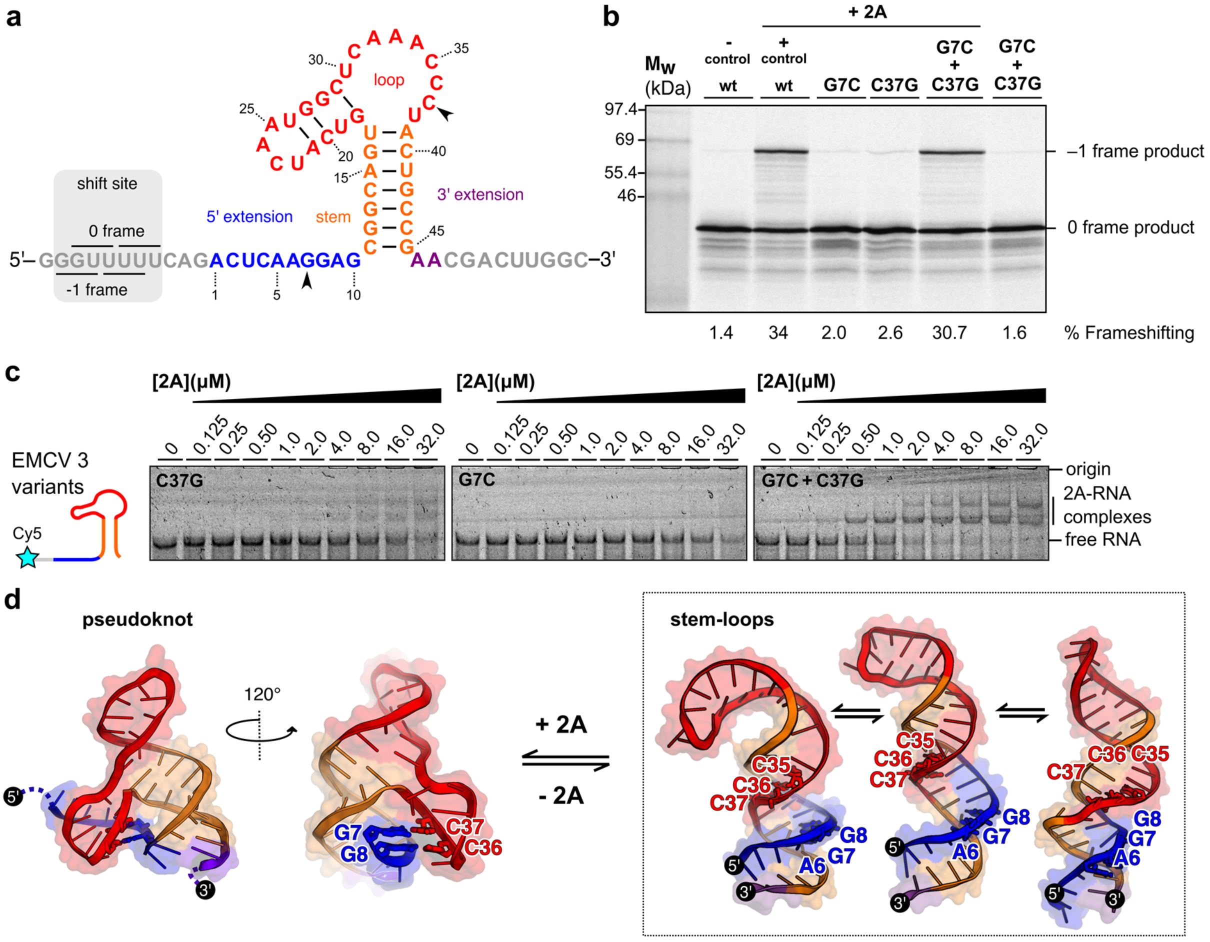

Supplementary Figure 3 – related to **Figure 2 and 3. Mutational analysis of the RNA stimulatory element suggests the existence of a pseudoknot-like conformation. a,** Schematic diagram showing numbered sequence of the EMCV 6 minimal PRF stimulatory element. **b,** Frameshifting assays showing evidence for a base-pairing interaction between G7 and C37. Individual G7C and C37C mutations reduce frameshifting to near-background levels. However, the double mutation (which would permit a compensatory C-G base-pair to form) restores frameshifting to wild-type levels. Representative autoradiogram from two independent experiments **c,** EMSA analyses showing that individual G7C and C37G mutations in the EMCV 6 RNA prevent 2A binding, but the double G7C+C37G mutation restores binding. Experiments were conducted with 50 nM Cy5-labelled EMCV 3 RNA variants and 2A concentrations between zero and 32 μM. Following non-denaturing electrophoresis, fluorescence was imaged using a Typhoon scanner. Showing representative gels from two independent experiments. **d,** Hypothetical equilibrium between several predicted stem-loops and alternate pseudoknot conformations, colour-coded as in **a**. The pseudoknot-like conformation involves base pairs between G7 and G8 in the 5′ extension and C36 and C37 in the loop (shown as sticks). These interactions are not maintained in any predicted stem-loop conformation. Predicted stem loops may involve 35 nt (core) or 49 nt (extended) structured

RNA. The predicted pseudoknot involves 40 nt structured RNA. Also see **Supplementary Table 6.**

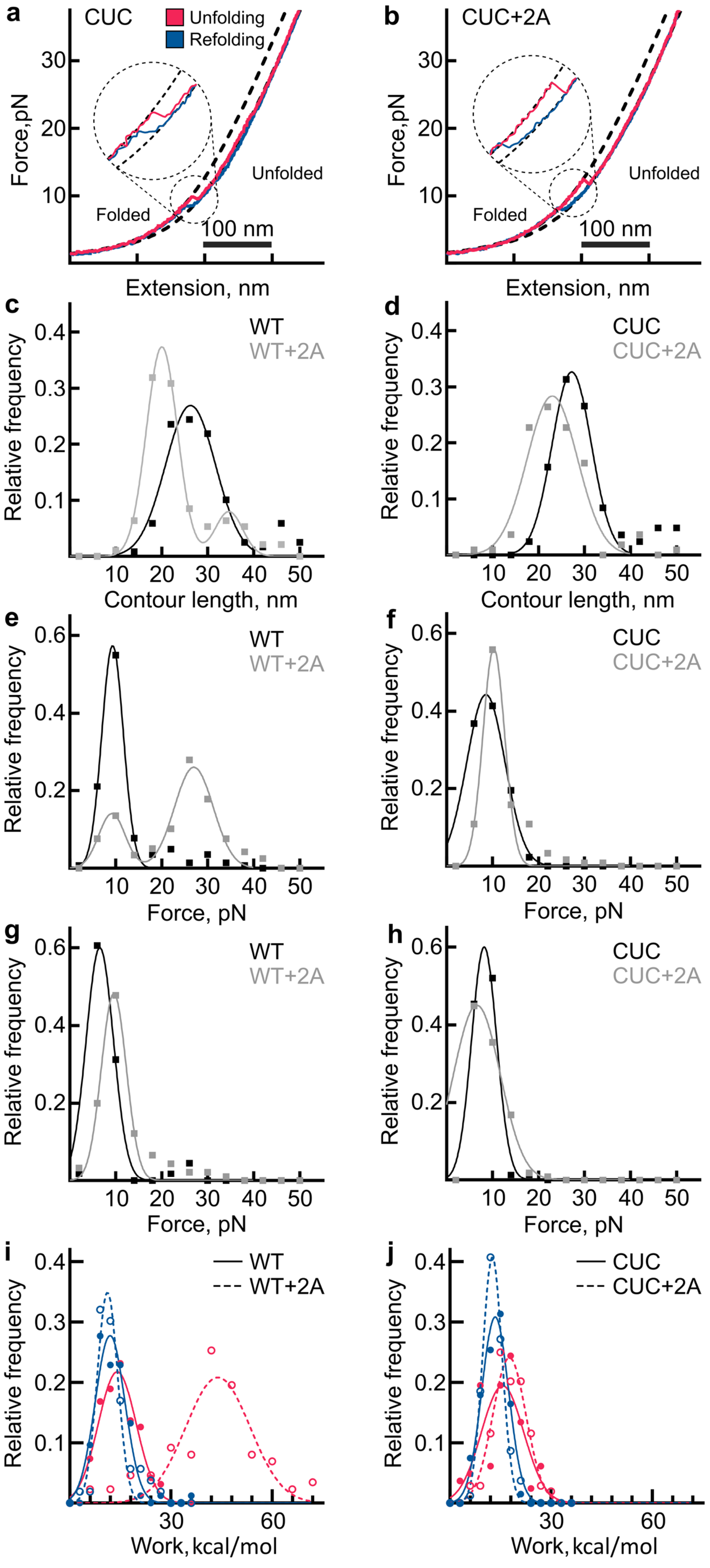
a
CUC
Unfolding
Refolding
Force,pN
Unfolded
Folded
100 nm
Extension, nm
b
CUC+2A
Unfolded
Folded
100 nm
Extension, nm
c
WT
WT+2A
Relative frequency
Contour length, nm
d
CUC
CUC+2A
Contour length, nm
e
WT
WT+2A
Force, pN
f
CUC
CUC+2A
Force, pN
g
WT
WT+2A
Force, pN
h
CUC
CUC+2A
Force, pN
i
WT
WT+2A
Work, kcal/mol
j
CUC
CUC+2A
Work, kcal/mol

Supplementary Figure 4 (previous page) – related to **Figure 3. Details of contour length, force and work distributions observed in optical tweezer experiments. a,** Representative force-distance curves of the unfolding (pink) and refolding (blue) transitions of the mutant CUC RNA element (n=85). **b,** Representative force-distance curves of the unfolding (pink) and refolding (blue) transitions of the mutant CUC RNA element in the presence of 300 nM 2A protein (n=109). **c** and **d**, distribution of the contour length changes in the absence (black) and presence (grey) of 2A for WT and CUC RNAs, respectively. Dots represent experimental data points while the line corresponds to the Gaussian fit. **e** and **f**, distribution of the unfolding forces in the absence (black) and presence (grey) of 2A for WT and CUC RNAs, respectively. **g** and **h**, distribution of the refolding forces in the absence (black) and presence (grey) of 2A for WT and CUC RNAs, respectively. **i** and **j**, distribution of unfolding (pink) and refolding (blue) work in the absence (solid) and presence (dashed) of 2A for WT and CUC, respectively.

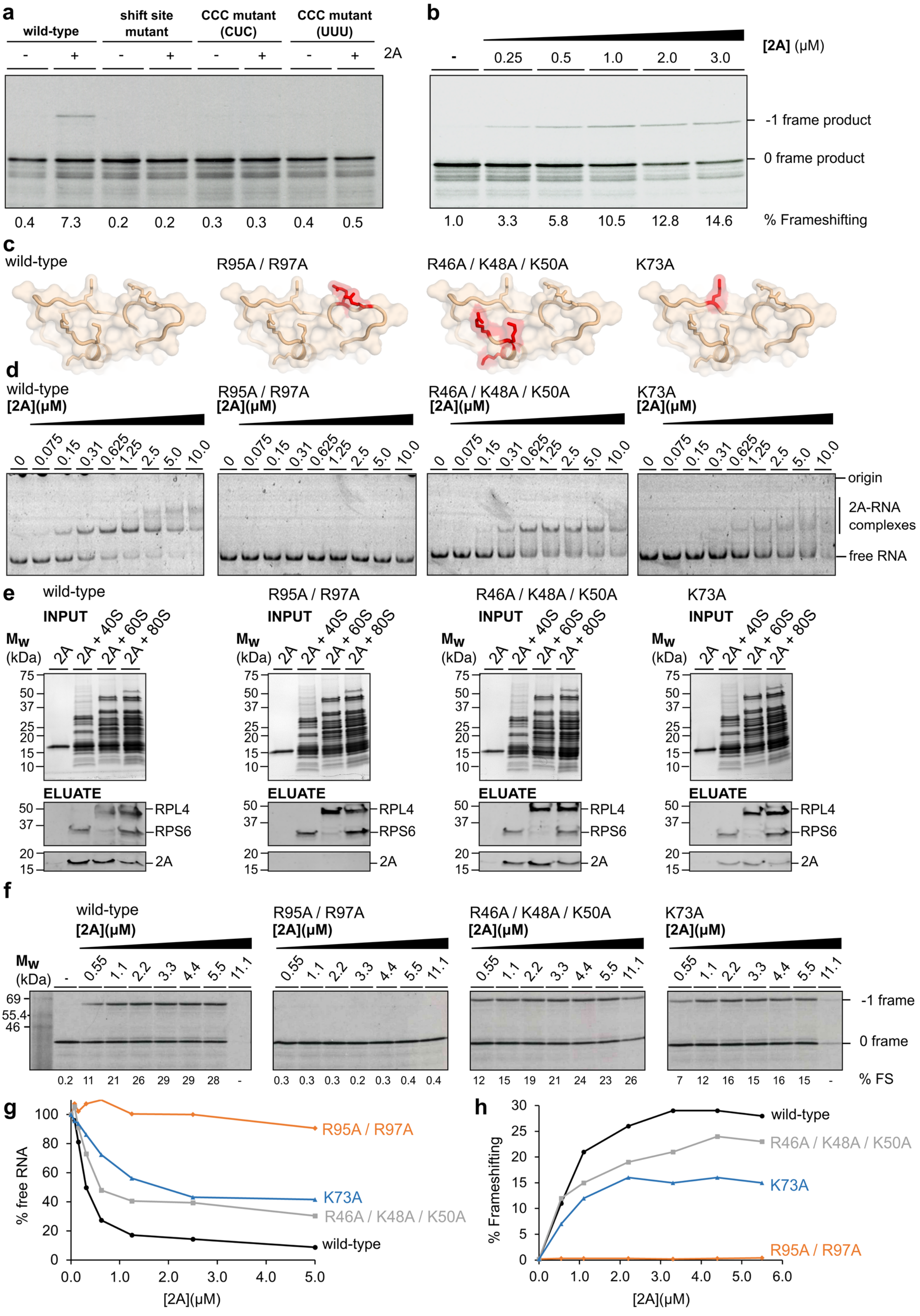

Supplementary Figure 5 – related to **Figures 5 and 6. Mutational analysis of 2A RNA-binding residues observed in the $70S_{IC}$-2A structure. Mutation of the 'arginine loop'**

disrupts binding to both stimulatory element RNA and mammalian ribosome subunits, and inhibits PRF. a, Frameshifting assay showing the reconstitution of 2A-dependent PRF in a prokaryotic *in vitro* translation system. Translation products were visualised by ^{35}S-Met autoradiography and % frameshifting was calculated following densitometry and correction for the number of methionines present in 0 frame and –1 frame products. Representative autoradiograms from two independent experiments. **b,** PRF efficiency in the prokaryotic system is proportional to 2A concentration. At high levels, 2A displays inhibitory effects on total translation. Data analysed as above. **c,** Mutagenesis of residues at the 2A RNA binding surface observed in the $70S_{IC}$-2A structure. The locations of mutations R95A/R97A, R46A/K48A/K50A and K73A are highlighted in red and shown as sticks. **d,** EMSA analyses showing effects of the above mutations on stimulatory element RNA binding, compared to a wild-type control. Panels ordered left-right, as in **a**. Experiments were conducted with 50 nM Cy5-labelled EMCV 6 RNA and 2A concentrations between zero and 10 µM. Following non-denaturing electrophoresis, fluorescence was imaged using a Typhoon scanner. Representative gels from two independent experiments. **e,** Gel-filtration experiments showing effects of the above mutations on eukaryotic ribosome binding, compared to a wild-type control. Panels ordered left-right, as in **a**. Excess 2A (2.5 µM) was incubated with 40S, 60S and 80S ribosomes (0.4 µM) prior to gel filtration chromatography using S200HR spin columns. In these experiments, 2A will only elute if bound to ribosomes. The input <*upper*> was analysed by 4-20% gradient SDS page and visualised by staining with Imperial protein stain. The eluate <*lower*> was analysed by western blot to detect 2A, RPS6 (small ribosomal subunit) and RPL4 (large ribosomal subunit). Experiments were performed once, and all mutant samples were processed at the same time as wild-type. **f,** Frameshift reporter assays showing the effects of the above mutations on *in vitro* translation in RRL. Translation products were visualised by ^{35}S-Met autoradiography and % frameshifting was calculated following densitometry and correction for the number of methionines present in 0 frame and –1 frame products. Representative autoradiograms from two independent experiments. **g,** Plot of % Free RNA vs. 2A concentration, based on densitometric quantification of EMSAs in **b**. **h,** Plot of % Frameshifting vs. 2A concentration, based on quantification reported in **d**.

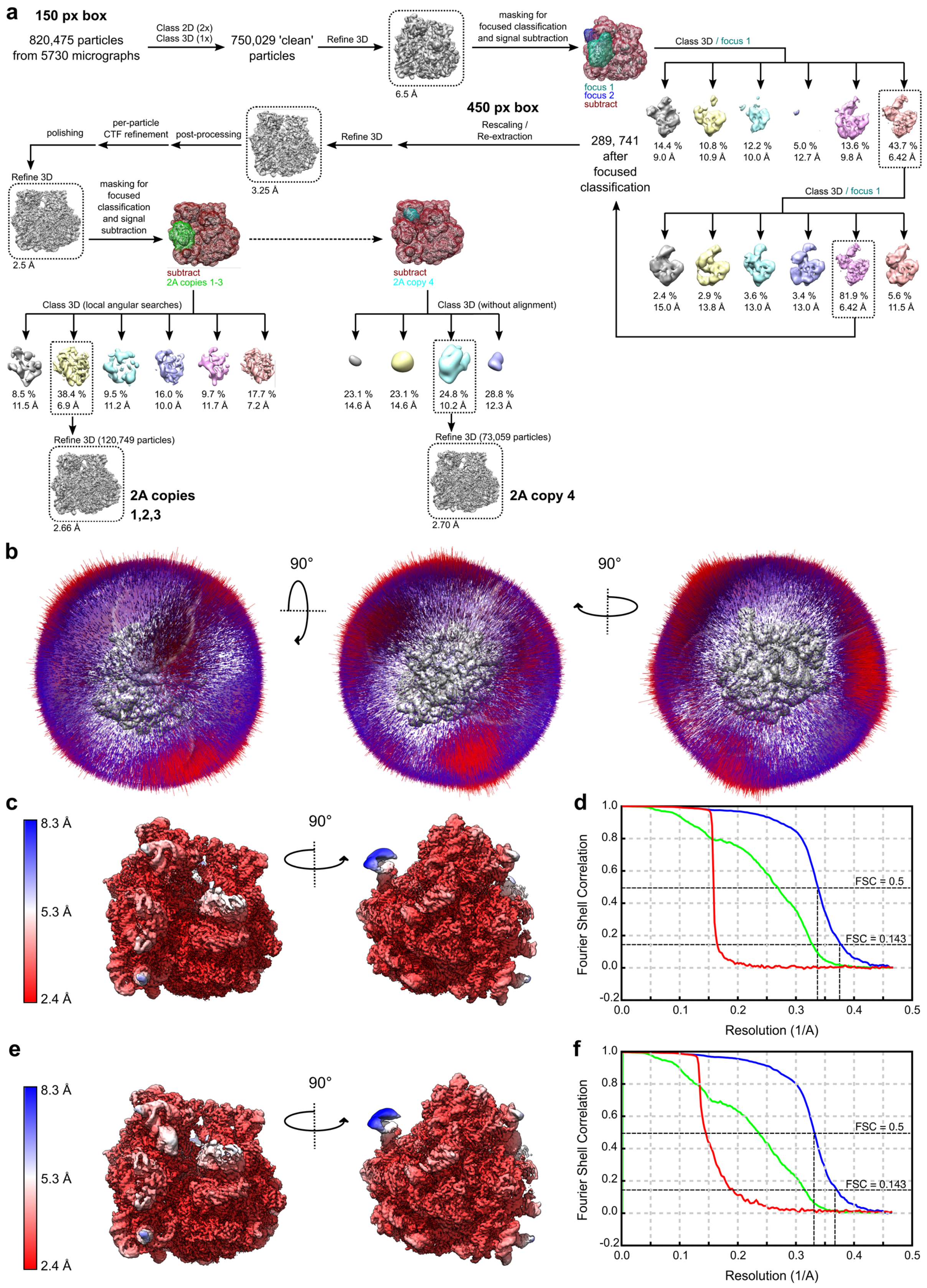

Supplementary Figure 6 – related to **Figures 5, 6 and 7. Details of cryo-EM image processing and map validation. a,** Schematic summary of steps in cryo-EM data processing.

b, Three orthogonal views showing the angular distribution of particles contributing to the final 3D reconstruction. This is shown for the highest-resolution Refine3D result i.e. immediately after particle polishing. **c,** Local-resolution map for the final reconstruction of 70S-2A$_3$. The surface is coloured by local resolution from red (highest; 2.4 Å) to blue (lowest; 8.3 Å). **d,** Gold-standard Fourier shell correlation (FSC) curve for the 70S-2A$_3$ map. Masked (blue), unmasked (green) and phase-randomised masked (red) plots are shown. **e,** Local-resolution map for the final reconstruction of 70S-2A$_4$, details as in **c**. Local resolution estimate for 2A4 is ~ 5 – 7 Å. **f,** Gold-standard Fourier shell correlation (FSC) curve for the 70S-2A$_4$ map. Details as in **d**.

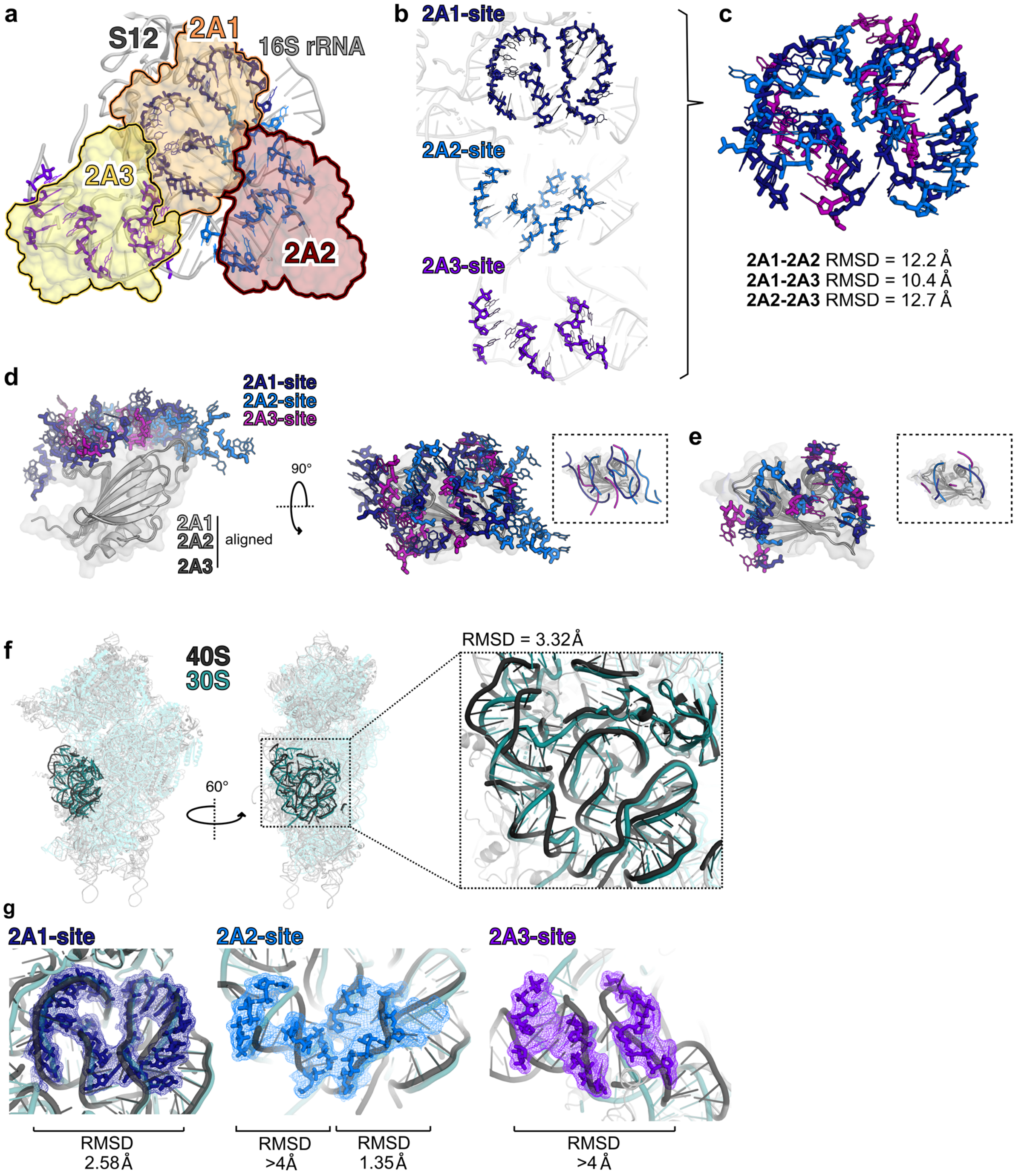

Supplementary Figure 7 – related to **Figure 5 and 6. Analysis of 2A binding sites observed on *E. coli* 16S rRNA. The 2A1 site is the most structurally conserved. a,** Diagram showing the binding sites of each 2A molecule (coloured sticks) on the 16S rRNA (grey cartoon). Binding sites were defined as rRNA residues within 8.0 Å distance of each 2A molecule, as determined by a PDBePISA analysis of molecular contacts. **b,** As above, showing each of the 2A binding sites separately (sticks), superimposed on local 16S rRNA (grey cartoon). **c,** Backbone superposition of the three sites fails to reveal a common binding motif. **d,** Superposition of binding sites (sticks) based on first aligning the three 2A molecules (grey ribbon diagrams). Two orthogonal views are shown. <*Inset*> cartoon representation of

RNA backbone shown in the top-down view. **e,** As in **d**, but trimmed to show spatial conservation (i.e. only residues within 3.0 Å of those in other binding sites. **f,** Structural superposition of 2A binding sites observed on *E. coli* 16S rRNA (30S, turquoise) with equivalent sites on *O. cuniculus* 18S rRNA (40S, black) The 30S 2A binding patch and equivalent 40S rRNA (black) are highlighted. <*Inset*> Close-up view of 2A binding sites after backbone superposition. **g,** Local alignment of each individual 2A binding site to nearest equivalent in 40S rRNA (black). Per site, deviation from the starting position in the 30S rRNA (green) was permitted. The binding site for 2A1 is the most structurally conserved between 30S and 40S.

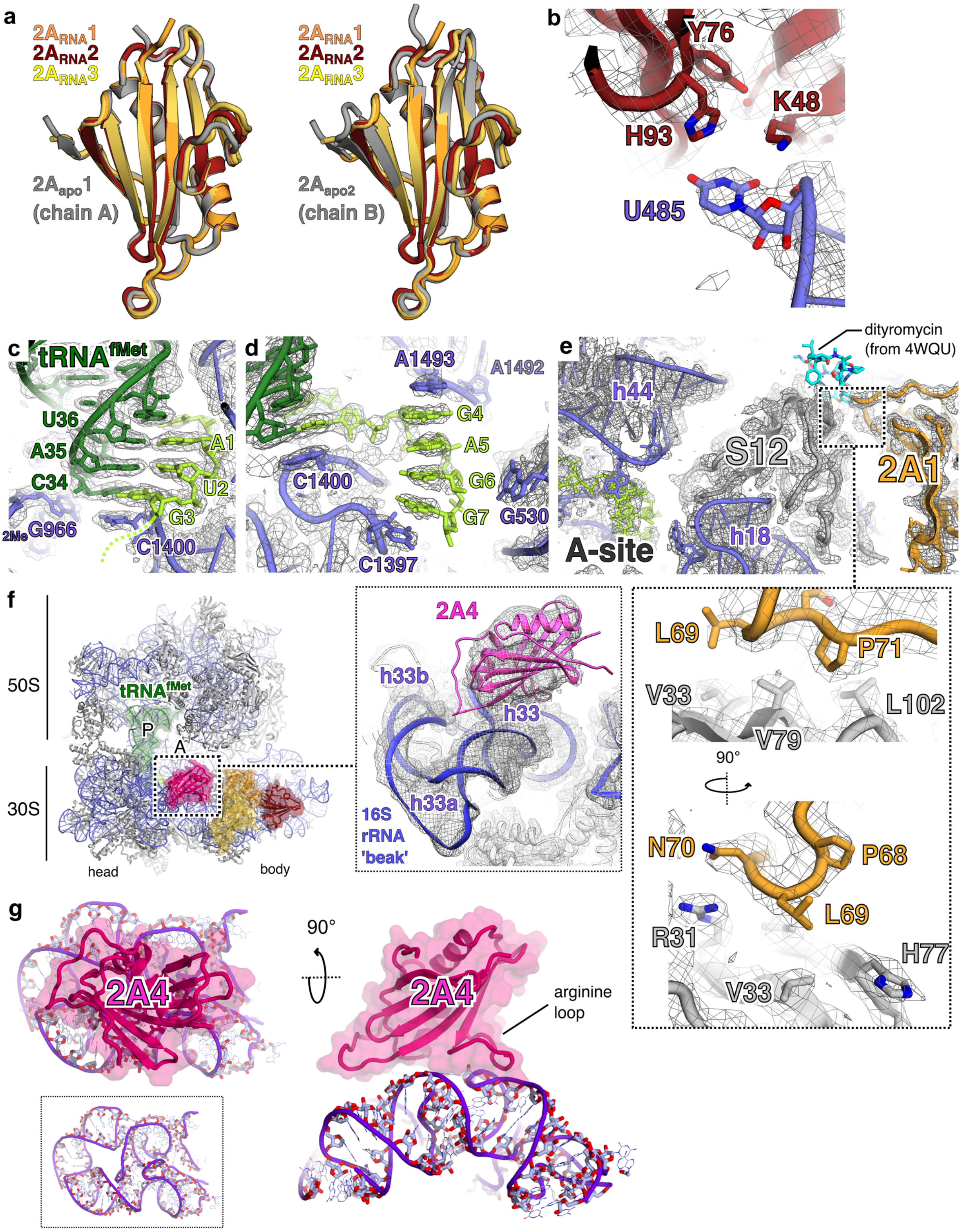

Supplementary Figure 8 – related to **Figure 5, 6 and 7. Structural details of the 70S decoding centre, 2A1 interaction with S12 and 2A4 binding site. a,** Comparison between conformations of 2A protein in RNA bound states (orange, red, yellow) and the two unliganded states observed by NCS in the crystal structure. The $2A_{apo}1$ conformation observed in chain A is most similar to the RNA-bound state. Structural alignments were performed by least-

squares superposition of the C_{α} backbone. **b,** Details of a base-specific interaction between U485 (helix 17 of 16S) and a pocket on the surface of 2A2 (red). **c,** Cryo-EM density at the P-site. Codon-anticodon pairing between the mRNA (lime) and the initiator $tRNA^{fMet}$ (dark green). The tRNA is in an undistorted P/P conformation as expected. **d,** Cryo-EM density at the A-site, coloured as in **b**. Additional 30S residues with roles in decoding are shown as sticks (purple). **e,** Details of a hydrophobic 2A1 interaction with ribosomal protein S12. The contact surface is on the factor-binding face of S12, away from the decoding centre. The binding site of antibiotic dityromycin on S12 (from 4WQU[4] [http://doi.org/10.2210/pdb4wqu/pdb]) is shown with blue sticks. **f,** Ribbon diagram of initiated 70S-mRNA-$tRNA^{fMet}$-2A complex showing the location of the fourth copy of 2A (pink) present in a smaller population of particles. Ribosome sites are labelled A and P. The initiator $tRNA^{fMet}$ (dark green), mRNA (lime), 2A1 (orange), 2A2 (red) and 2A3 (yellow) are also shown. <*Inset*> Section of the 70S-$2A_4$ local resolution map showing electron density at the 2A4 binding site. 2A4 binds to the 3′ major ‘beak’ domain of the 16S rRNA present in the 30S ‘head’, via electrostatic interactions with the ribose phosphate backbone of helices 33 and 34. **g,** Details of 2A4 interaction with 16S rRNA (purple) in two orthogonal views.

Additional Data [http://dx.doi.org/10.17632/gkpwngy65h.2] – related to **Figures 2 and 4. Capillary scans and thermophoretic time-traces of microscale thermophoresis (MST) measurements of binding between 2A and RNA. a,** EMCV RNA 1, **b,** EMCV RNA 2, **c,** EMCV RNA 3, **d,** EMCV RNA 4, **e,** EMCV RNA 5, **f,** EMCV RNA 5, **g,** EMCV RNA 6, **h,** 30S, **i,** 50S, **j,** 40S, **k,** 60S, **l,** 70S and **m,** 70S IC. The protein was fluorescently labelled, while unlabelled ligands were added at different concentrations. The grey boxes in the capillary scans mark 20% above and below the average peak fluorescence, the acceptable limit of deviations across the fluorescence scans. Blue and red boxes in the time-course traces represent the temperature jump and MST-on time (10s), respectively. In all cases, there is no adsorption of the labelled protein to the capillaries. See Fig. 2 and Fig. 4 for the resulting binding curves.

Supplementary Tables

Supplementary Table 1 – Crystallographic data collection and refinement. Related to **Figure 1.**

	Native (PDB 7BNY)	SeMet derivative		
**Data collection**				
Space group	$P6_222$			
Cell dimensions				
a, *b*, *c* (Å)	91.5, 91.5, 316.5			
α, β, γ (°)	90.0, 90.0, 120.0			
		Peak	*Inflection*	*Remote (High E)*
Wavelength (Å)	0.97958	0.97965	0.97974	0.97635
Resolution (Å)	43.91–2.62 (2.67–2.62)	76.53–2.91 (2.96–2.91)	79.36–3.08 (3.13–3.08)	79.0–2.98 (3.03–2.98)
R_{pim}	0.053 (0.640)	0.062 (0.521)	0.067 (0.548)	0.048 (0.473)
$I / \sigma I$	11.5 (1.0)	9.9 (1.2)	9.3 (1.1)	12.9 (1.2)
Completeness (%)	100.0 (99.8)	99.7 (99.2)	99.9 (99.6)	99.6 (99.0)
Anomalous	-	99.2 (98.8)	99.3 (99.2)	99.1 (98.7)
Redundancy	18.9 (19.4)	18.6 (17.2)	18.6 (19.8)	37.4 (38.5)
Anomalous	-	10.4 (9.3)	10.5 (10.7)	21.0 (20.9)
$CC_{1/2}$	0.998 (0.728)	0.997 (0.804)	0.997 (0.822)	0.999 (0.834)
CC_{anom}	-	0.400 (0.024)	0.167 (0.000)	0.314 (0.002)
**Refinement**				
Resolution (Å)	43.91–2.62 (2.71–2.62)			
No. reflections	24589 (2375)			
R_{work} / R_{free}	0.2254 / 0.2511			
No. atoms				
Protein	4404			
Ligand/ion	45			
Water	147			
B-factors				
Protein	75.0			
Ligand/ion	110.0			
Water	64.9			
R.m.s deviations				
Bond lengths (Å)	0.003			
Bond angles (°)	0.741			

*Data were recorded from a single native crystal and a single crystal of selenomethionine-derivatised protein (SeMet). *Values in parentheses are for the highest-resolution shell.

Supplementary Table 2 - Summary of dissociation constants (K_D) measured by microscale thermophoresis with various 2A interaction partners. Related to **Figures 2, 4 and Supplementary Figure 2.**

	K_D (LABELLED 2A)	K_D (LABELLED RNA)
EMCV 1	360 ± 34 nM	490 ± 45 nM
EMCV 2	No binding	No binding
EMCV 3	40 ± 2 nM	31 ± 4 nM
EMCV 4	No binding	No binding
EMCV 5	> 4 µM (does not reach saturation)	> 4 µM (does not reach saturation)
EMCV 6	70 ± 14 nM	50 ± 12 nM
40S	10 ± 2 nM (apparent)	N/A
60S	> 1000 nM (apparent; does not reach saturation)	N/A
30S	4 ± 1 nM (apparent)	N/A
50S	> 500 nM (apparent)	N/A
70S	49 ± 13 nM (apparent)	N/A
70S IC	> 60 nM (apparent; does not reach saturation)	N/A

Supplementary Table 3 – Summary of experimental OT results and predicted mfold data for EMCV WT and CUC RNAs in the absence or presence of 2A. Results were determined by gaussian fitting of the distribution histograms of obtained data (number of tethers used in the experiments was >15, and number of force trajectories range between 85-120). Related to **Figure 3 and Supplementary Figure 4.** Uncertainties represent standard deviations.

	WT	WT + 2A	CUC	CUC + 2A
Contour length change, nm	26.3±5.4	20.1±3.4	27.2±4.3	23.0±5.5
Unfolding force, pN	9.3±2.3	9.2±2.9<br>27.0±4.2	8.6±4.2	10.3±2.4
Refolding force, pN	6.5±3.0	9.6±2.7	8.2±2.7	6.6±5.1
Gibbs free energy (mfold), kcal/mol	-14.0±0.7 (SL),<br>-16.2±0.8<br>(extended SL)		-14.0±0.7 (SL),<br>-16.2±0.8<br>(extended SL)	
Gibbs free energy (experiment), kcal/mol	-13.6±4.6*<br>-11.6±0.9**	-<br>-26.5±8.7**	-14.5±4.7*<br>-11.0±1.6**	-15.5±5.0*<br>-12.9±1.2**

*Calculated by applying Crook's fluctuation theorem to unfolding/refolding work distributions. Uncertainty represents standard deviation.
**Calculated by applying Jarzynski's equality. Uncertainty represents root of mean square error.

Of note, free energy values of the CUC and WT (without 2A) RNAs were in good agreement regardless of the calculation method employed.

Supplementary Table 4 – Cryo-EM data collection, processing, refinement and validation. Related to **Figures 5–7, Supplementary Figure 6 and 7**.

	70SIC-2A complex (EMDB-12635) (PDB 7NWT)
**Data collection and processing**	
Magnification	75,000x
Voltage (kV)	300
Electron exposure (e–/Å^2)	54.4
Defocus range (μm)	–1.2, –1.5, –1.8, –2.1, –2.4, –2.7, –3.0
Pixel size (Å)	1.07
Symmetry imposed	C1
Initial particle images (no.)	820,475
Final particle images (no.)	120,749
Map resolution (Å)	2.66
FSC threshold	0.143
Map resolution range (Å)	2.66 – 8.3
**Refinement**	
Initial model used (PDB code)	5MDZ
Model resolution (Å)	2.66
FSC threshold	0.143
Map sharpening *B* factor (Å^2)	-88.5
Model composition	
Non-hydrogen atoms	149932
Protein residues	6355
Nucleic acid residues	4641
Ligands	439
B factors (Å^2)	
Protein	53.87
Nucleic Acid	49.01
Ligand	34.31
R.m.s. deviations	
Bond lengths (Å)	0.011
Bond angles (°)	0.830
**Validation**	
MolProbity score	1.73
Clashscore	4.84
Poor rotamers (%)	2.95
Ramachandran plot	
Favored (%)	97.41
Allowed (%)	2.58
Disallowed (%)	0.02

Supplementary Table 5 – Oligonucleotide sequences. Related to **Methods**.

RNAs	SOURCE / REF
EMCV 1 used in EMSA and MST assays:<br>5' –<br>ACUCAAGGAGCGGCAGUGUCAUCAAUGGCUCAAACCCUACUGCCGAAC<br>GACUUG – 3'	Integrated DNA Technologies (IDT)
EMCV 2 used in EMSA and MST assays:<br>5' –<br>AGCGGCAGUGUCAUCAAUGGCUCAAACCCUACUGCCGAACGACUUG –<br>3'	Integrated DNA Technologies (IDT)
EMCV 3 used in EMSA and MST assays:<br>5' –<br>GGGUUUUUCAGACUCAAGGAGCGGCAGUGUCAUCAAUGGCUCAAACCC<br>UACUGCCGAA – 3'	Integrated DNA Technologies (IDT)
EMCV 3a used in EMSA<br>5' –<br>GGGUUUUUCAGACUCAAGGAGCGGCAGUGUCAUCAAUGGCUCAAACC<br>GUACUGCCGAA – 3'	Integrated DNA Technologies (IDT)
EMCV 3b used in EMSA<br>5' –<br>GGGUUUUUCAGACUCAACGAGCGGCAGUGUCAUCAAUGGCUCAAACCC<br>UACUGCCGAA – 3'	Integrated DNA Technologies (IDT)
EMCV 3c used in EMSA<br>5' –<br>GGGUUUUUCAGACUCAACGAGCGGCAGUGUCAUCAAUGGCUCAAACCG<br>UACUGCCGAA – 3'	Integrated DNA Technologies (IDT)
EMCV 4 used in EMSA and MST assays:<br>5' – AGCGGCAGUGUCAUCAAUGGCUCAAACCCUACUGCCGAA – 3'	Integrated DNA Technologies (IDT)
EMCV 5 used in EMSA and MST assays:<br>5' –<br>GGGUUUUUCAGACUCAAGGAGCGGCAGUCAAACCCUACUGCCGAACGA<br>CUUG – 3'	Integrated DNA Technologies (IDT)
EMCV 6 used in EMSA and MST assays:<br>5' –<br>ACUCAAGGAGCGGCAGUGUCAUCAAUGGCUCAAACCCUACUGCCGAA –<br>3'	Integrated DNA Technologies (IDT)
70S_IC transcribed mRNA (used in 70S IC preparation):<br>5' –<br>GGGAAUUCAAAAAUUGUUAAGAAUUAAGGAGAUAUACAUAUGGAGGUU<br>UUUAUCACUCAAGGAGCGGCAGUGUCAUCAAUGGCUCAAACCCUACUG<br>CCGAACGACUUGGCCAGATCT – 3'	This work
EMCV CCC OT ssRNA region of the OT construct (used in OT experiments):<br>5'–<br>GAGGUUUUUAUCCUCAAGGAGCGGCAGUGUCAUCAAUGGCUCAAACCC<br>UACUGCCGAACGACUUGGACUUAAUUUAAAAAAAAAAAAAAAAAA<br>– 3'	This work

EMCV CUC OT ssRNA region of the OT construct (used in OT experiments):<br>5' –<br>GAGGUUUUUAUCCUCAAGGAGCGGCAGUGUCAUCAAUGGCUCAAACUC<br>UACUGCCGAACGACUUGGACUUAAUUUAAAAAAAAAAAAAAAAAAA – 3'	This work

**DNA Oligonucleotides**	**SOURCE / REF**
E2A_F1 primer (used for cloning EMCV 2A):<br>5' – AATTCATATGAGCCCCAACCCTTTGGATGTC – 3'	Sigma-Aldrich<br>VC00021
E2A_R1 primer (used for cloning EMCV 2A):<br>5' – AATTGGATCCCCCGGGATTGGTCTCGACATC – 3'	Sigma-Aldrich<br>VC00021
E2A_F2 primer (used to clone EMCV 2A 9-136 construct):<br>5' – AATTCATATGAAAACTTACCCAACTCTGCACAT – 3'	Sigma-Aldrich<br>VC00021
E2A_R2 primer (used to clone EMCV 2A 9-136 construct]):<br>5' – AATTGGATCCTCATTAGTGGATCAAAAGATCTGAAAAGTATCCT – 3'	Sigma-Aldrich<br>VC00021
E2A_mut_F1 mutagenesis primer (used to introduce 2A mutant C111S):<br>5' – AAAGAGTGGAGGACTTCTGAAGAGAATGTTTTT – 3'	Sigma-Aldrich<br>VC00021
E2A_mut_R1 mutagenesis primer (used to create 2A C111S):<br>5' – AAAAACATTCTCTTCAGAAGTCCTCCACTCTTT – 3'	Sigma-Aldrich<br>VC00021
E2A_mut_F2 mutagenesis primer (used to create 2A R46A/K48A/K50A):<br>5' –<br>AACACATGCAGAAGTGGTGCTTGCGTCAGCGACAGCACAAATCAGCTTC<br>CTGAGCAA – 3'	Sigma-Aldrich<br>VC00021
E2A_mut_R2 mutagenesis primer (used to create 2A R46A/K48A/K50A):<br>5' –<br>TTGCTCAGGAAGCTGATTTGTGCTGTCGCTGACGCAAGCACCACTTCTG<br>CATGTGTT – 3'	Sigma-Aldrich<br>VC00021
E2A_mut_F3 mutagenesis primer (used to create 2A K74A):<br>5' – CACCGTTAAACCCCTGGGCGAGCACATATCAGGCAGT – 3'	Sigma-Aldrich<br>VC00021
E2A_mut_R3 mutagenesis primer (used to create 2A K74A):<br>5' – ACTGCCTGATATGTGCTCGCCCAGGGGTTTAACGGTG – 3'	Sigma-Aldrich<br>VC00021
SII_F cloning oligonucleotide (used to insert StrepII-tag into 2A_pGEX6P1):<br>5' –<br>GATCCTGGTCACATCCGCAGTTTGAAAAAGGTAGCGCAGGTAGTGCAGC<br>AGGTAGCGGTGCAGGTTGGAGCCACCCTCAGTTTGAGAAAG – 3'	Sigma-Aldrich<br>VC00021
SII_R cloning oligonucleotide (used to insert StrepII-tag into 2A_pGEX6P1):<br>5' –<br>GATCCTTTCTCAAACTGAGGGTGGCTCCAACCTGCACCGCTACCTGCTG<br>CACTACCTGCGCTACCTTTTTCAAACTGCGGATGTGACCAG – 3'	Sigma-Aldrich<br>VC00021
pdluc/EMCV reporter construct sequence (used in frameshift assays):<br>5'-<br>AAGACAACGGCCGGTTTTTCAGACTCAAGGAGCGGCAGTGTCATCAATG<br>GCTCAAACCCTACTGCCGAACGACTTGGCCAGCAAAcgTATGGGATCAGC<br>CTTTAC– 3'	Napthine et al., 2017
Transcription template forward primer (used in 70S IC preparation):<br>5' – GTTGTGTGGAATTGTGAGCGGAT– 3'	Microsynth
Transcription template reverse primer (used in 70S IC preparation):	Microsynth

5’ – GGCCAAGTCGTTCGG – 3’	
5' handle T7 forward PCR primer (used to generate OT 5’ handle):<br>5’ – CTTAATACGACTCACTATAGGTCCTTTCTGTGGACGCC – 3’	Microsynth
5’ handle reverse PCR primer (used to generate OT 5’ handle and clone short ssRNA control construct):<br>5’ – CATAAATACCTCTTTACTAATATATATACCTTCGTAAGCTAGCGT – 3’	Microsynth
5’ handle short single stranded control PCR primer (used to generate OT 5’ handle for short ssRNA control and clone no ssRNA control construct):<br>5’-<br>GTAGCTGTCGAGCTCCTGCGAAGAGCGCAACCATGTTATCCAGTGAG– 3’	Microsynth
3’ handle forward PCR primer (used to generate OT 3’ handle and to clone both of the control constructs):<br>5’ – ATCCTGCAACCTGCTCTTCGCCAG – 3’	Microsynth
3’ handle reverse 5’labeled with **digoxigenin** PCR primer (used to generate OT 3’ handle):<br>5’ –[Dig]-GTCAAAGTGCGCCCCGTTATCC – 3’	Microsynth
CUC mutant forward cloning oligonucleotide (used to insert the point mutation of CCC motif):<br>5’ – CTCTACTGCCGAACGACTTGGCC – 3’	Microsynth
CUC mutant reverse cloning oligonucleotide (used to insert the point mutation of CCC motif):<br>5’ – TTTGAGCCATTGATGACACTGCCG – 3’	Microsynth

The name of each oligonucleotide is highlighted in bold italics, as referred to in **Methods**.

Supplementary Table 6 – Predicted secondary structures of the EMCV PRF RNA element. Related to **Figure 3** and **Supplementary Figure 4**.

**Structure**	**Expected contour length, nm / nt**	**Predicted secondary structure**
Primary sequence	55.5 / 94	GAGGUUUUUAUCCUCAAGGAGCGGCAGUGUCAUCAAUGG CUCAAACCCUACUGCCGAACGACUUGGACUUAAUUUAAAA AAAAAAAAAAAAAAA
Stem-loop	20.7 / 35	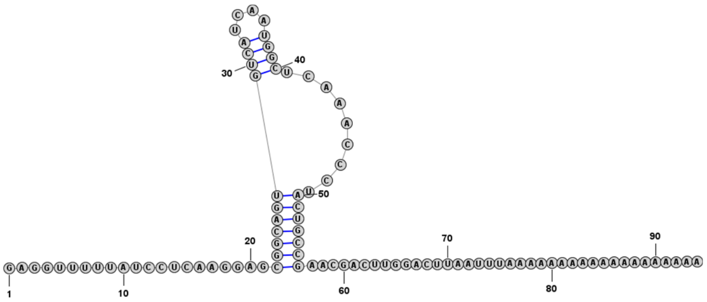
Extended stem-loop	28.9 / 49	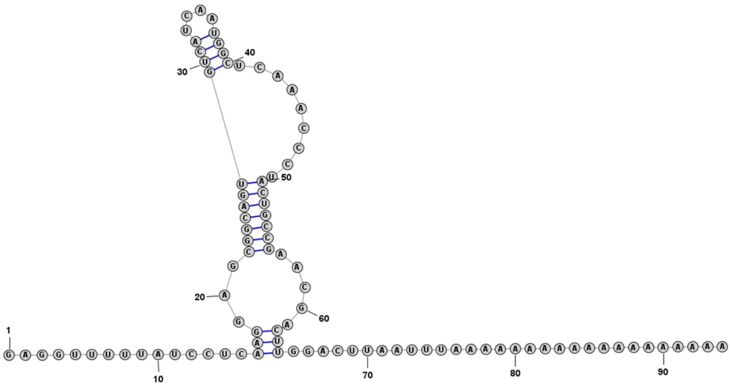
Pseudoknot	23.5 / 40	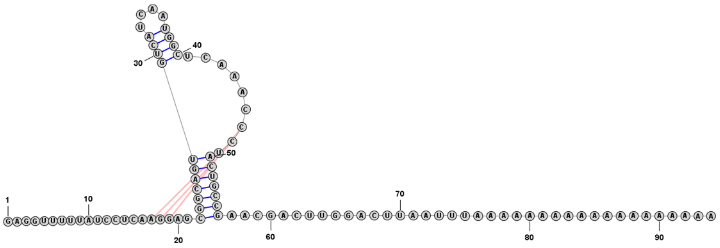

Source Data for Supplementary Figures (uncropped images, autoradiograms, gel scans, phosphorimager scans)

Supplementary Figure 2 – panel A (fluorescent gels)

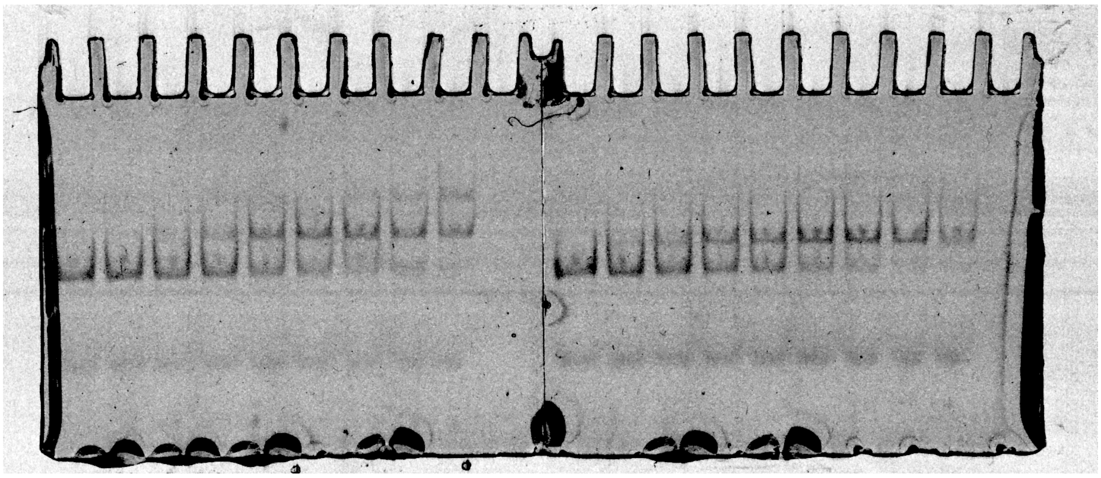

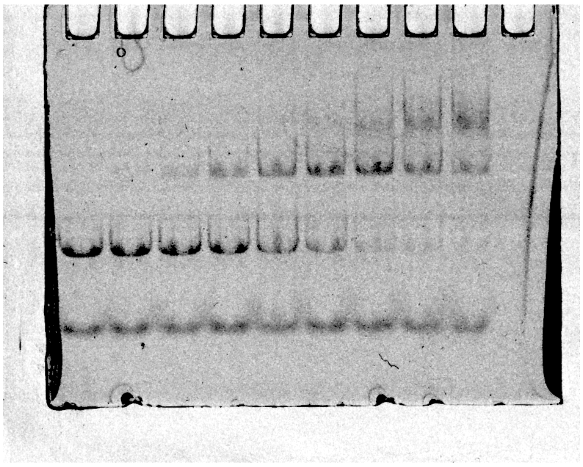

Supplementary Figure 2 – panel E (phosphorimager scan)

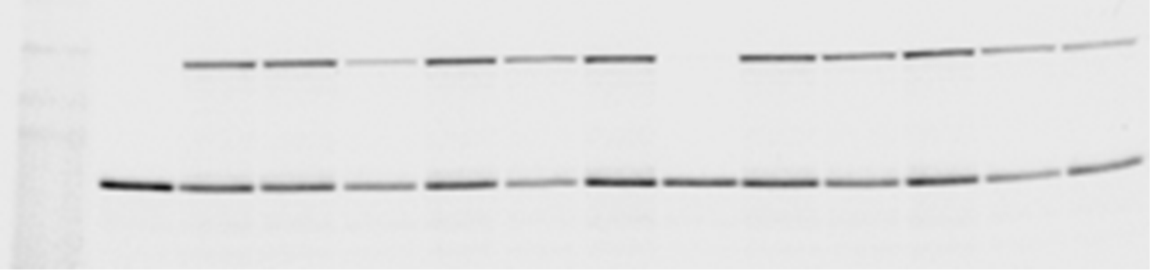

Supplementary Figure 3 – panel B (phosphorimager scan)

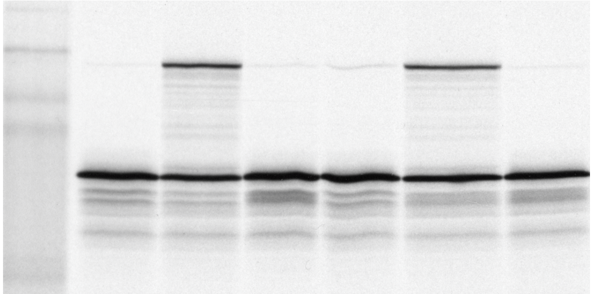

Supplementary Figure 3 – panel C (fluorescent gels)

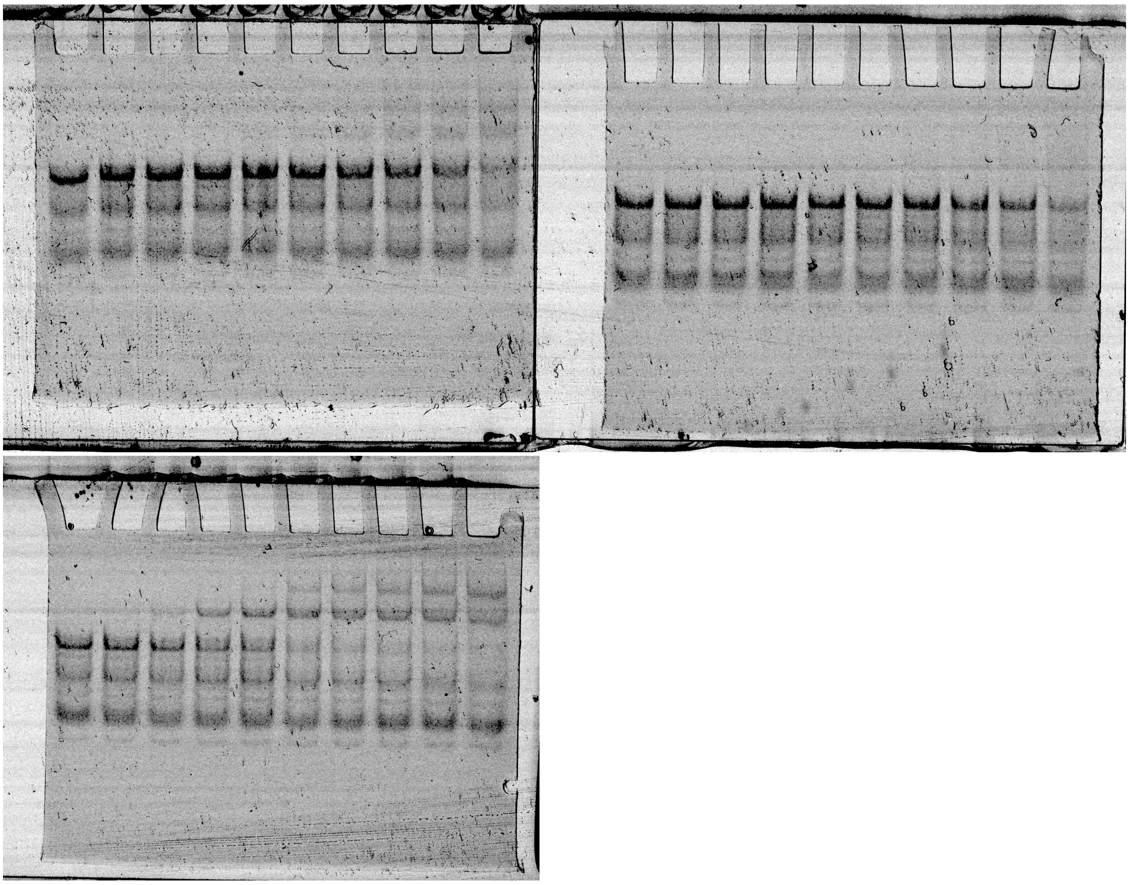

Supplementary Figure 5 – panel A (phosphorimager scan)

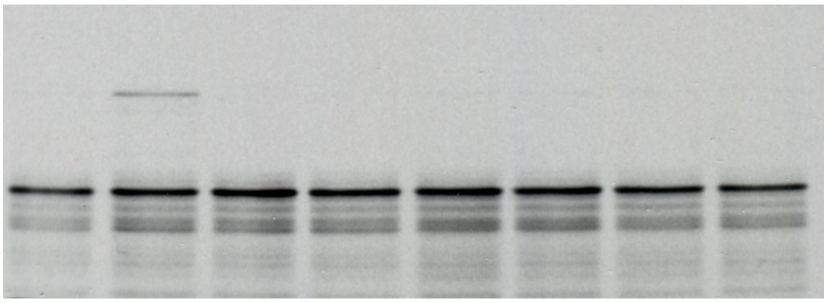

Supplementary Figure 5 – panel B (phosphorimager scan)

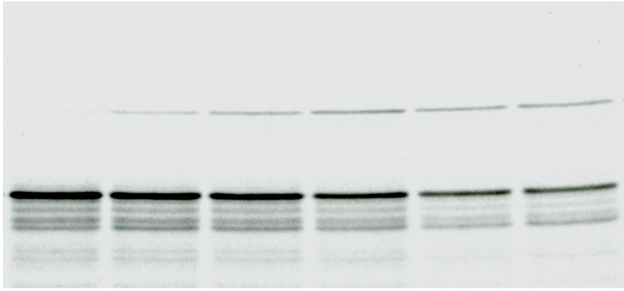

Supplementary Figure 5 – panel D (fluorescent gels)

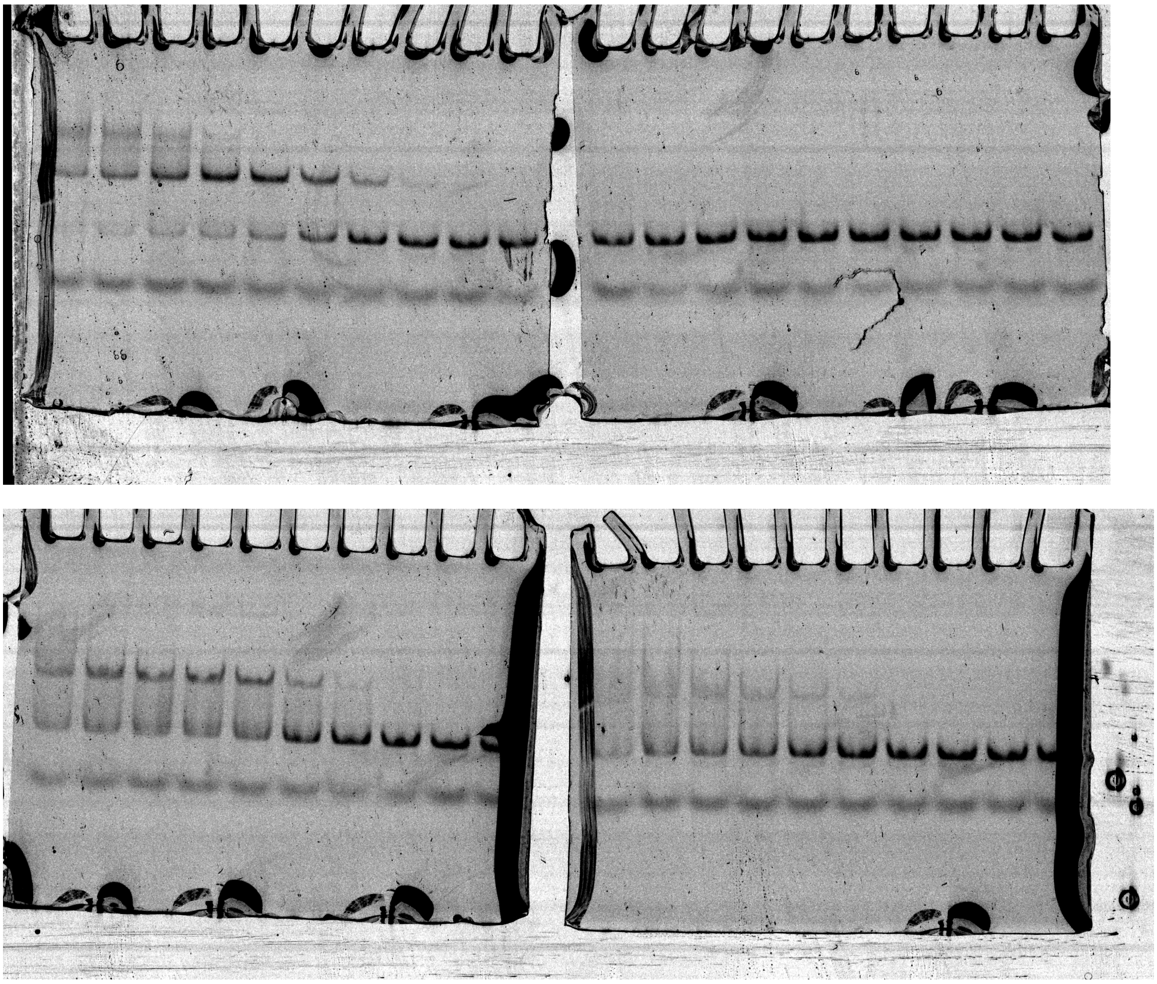

Supplementary Figure 5 – panel E (Imperial-stained input gels. The molecular weight ladders are the Precision Plus Protein Dual Color Standards [BioRad, 1610374])

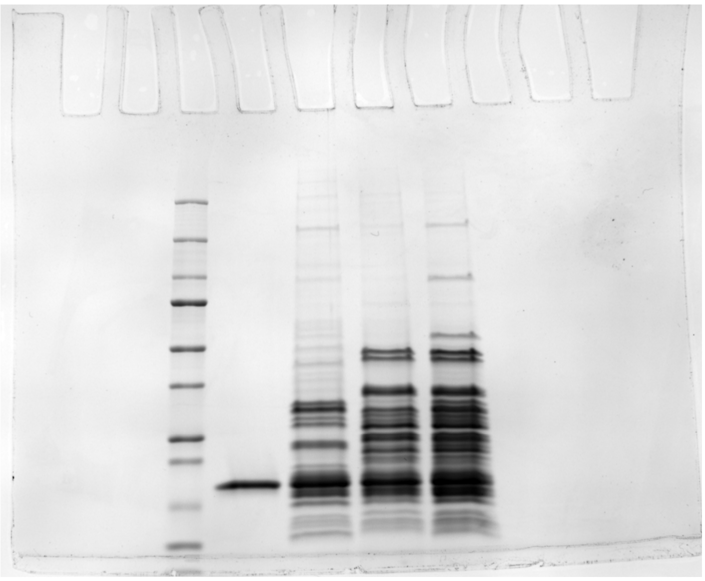

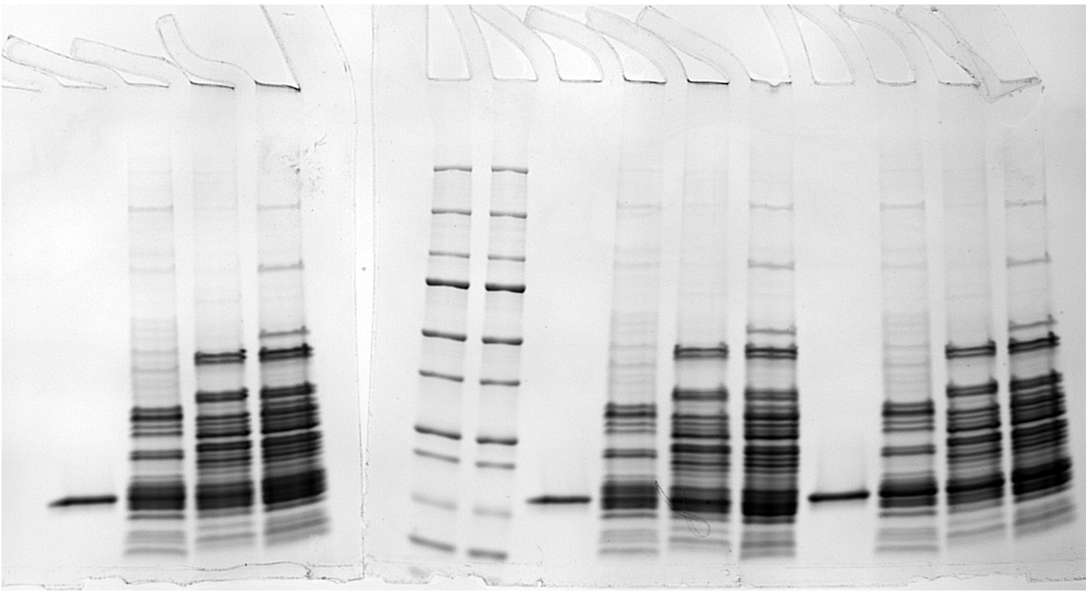

Supplementary Figure 5 – panel E (LI-COR fluorescent scans of eluate blots, green channel – anti-S6 and anti-L4 ribosomal proteins; red channel anti-2A. The molecular weight ladders are the Precision Plus Protein Dual Color Standards [BioRad, 1610374])

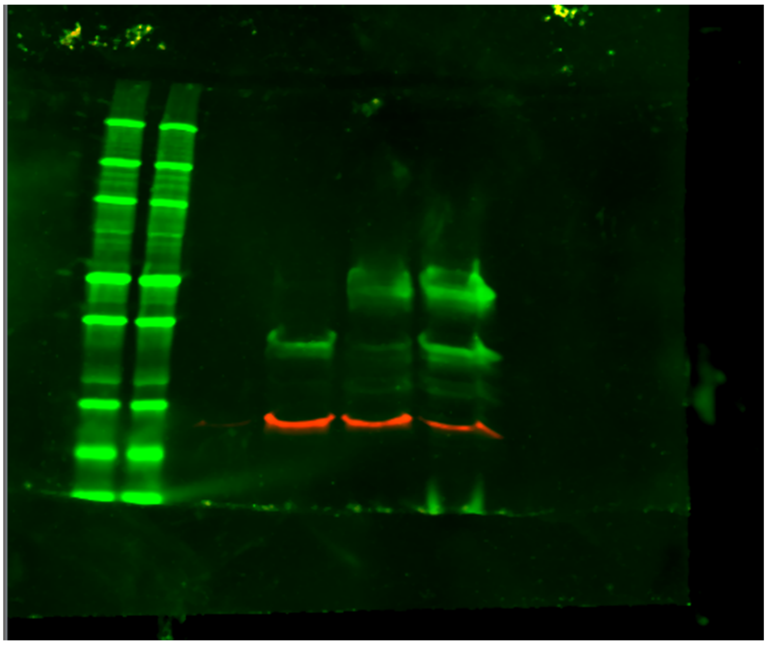

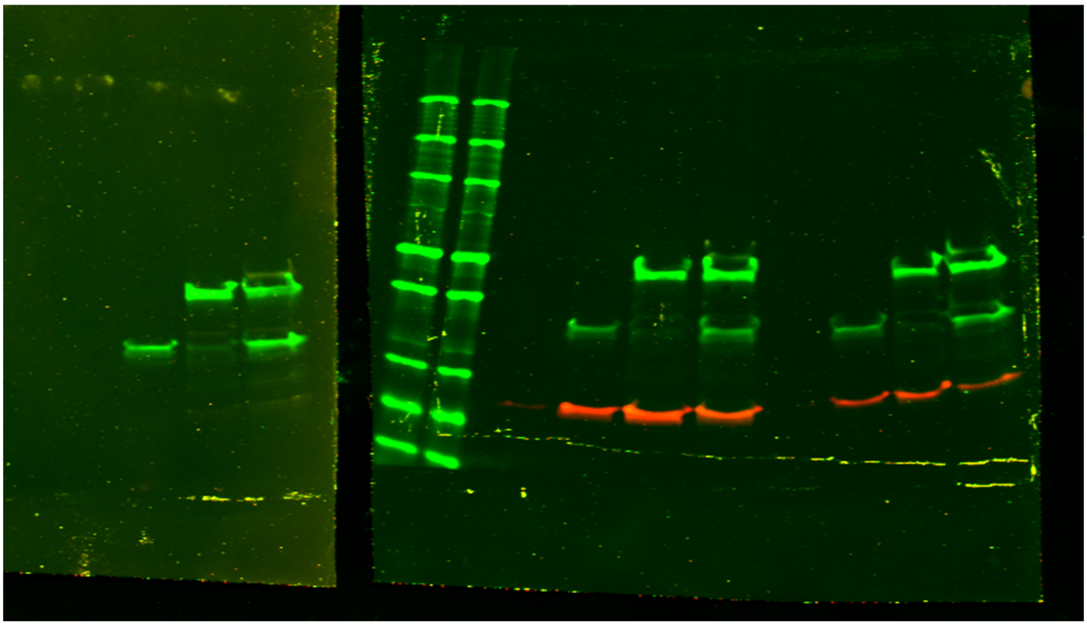

Supplementary Figure 5 – panel F (autoradiograms)

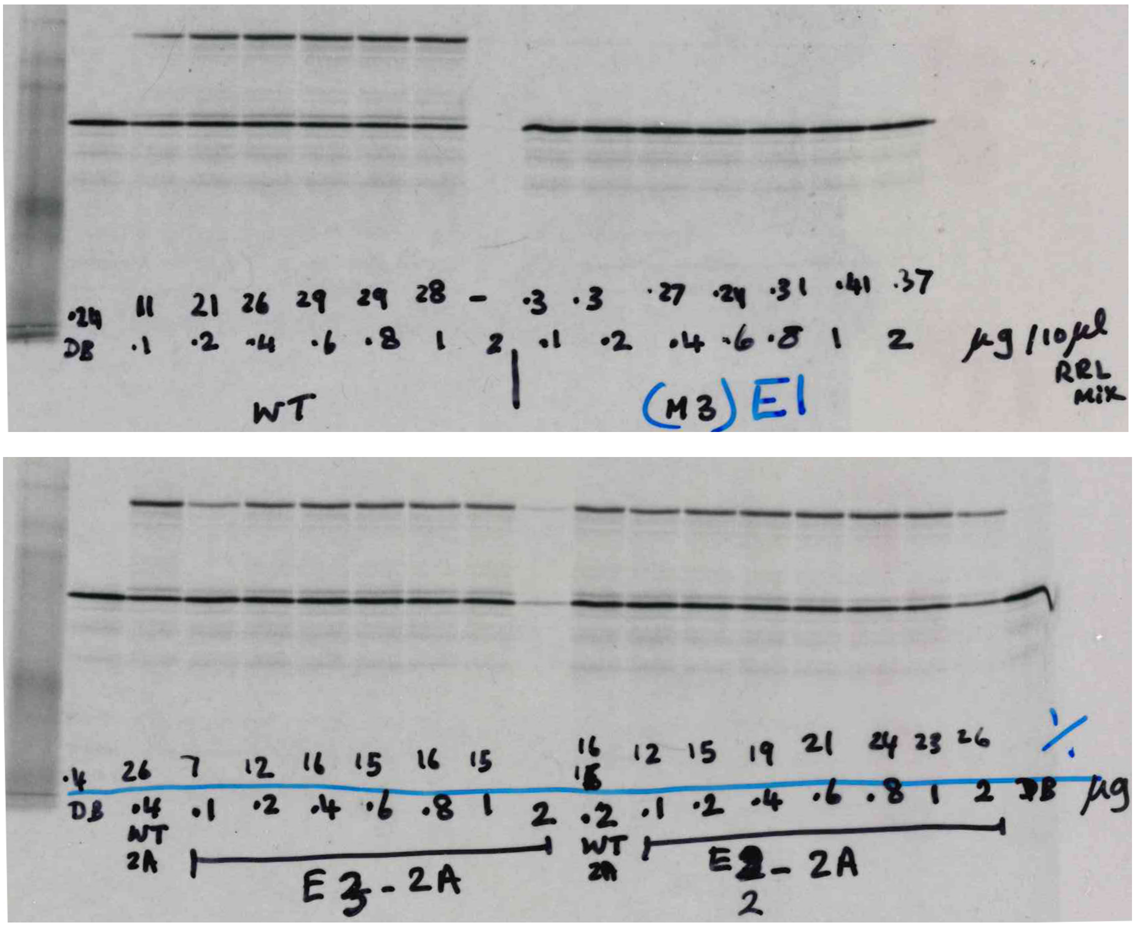

Supplementary References

1. Svitkin, Y.V., Hahn, H., Gingras, A.C., Palmenberg, A.C. & Sonenberg, N. Rapamycin and wortmannin enhance replication of a defective encephalomyocarditis virus. *J Virol* **72**, 5811-9 (1998).
2. Petty, R.V., Basta, H.A., Bacot-Davis, V.R., Brown, B.A. & Palmenberg, A.C. Binding interactions between the encephalomyocarditis virus leader and protein 2A. *J Virol* **88**, 13503-9 (2014).
3. Groppo, R., Brown, B.A. & Palmenberg, A.C. Mutational analysis of the EMCV 2A protein identifies a nuclear localization signal and an eIF4E binding site. *Virology* **410**, 257-67 (2011).
4. Lin, J., Gagnon, M.G., Bulkley, D. & Steitz, T.A. Conformational changes of elongation factor G on the ribosome during tRNA translocation. *Cell* **160**, 219-27 (2015).
