# Supplementary material for: Structural and molecular basis for Cardiovirus 2A protein as a viral gene expression switch
Source: Nat Commun. 2021 Dec 9;12:7166. doi: 10.1038/s41467-021-27400-7 (PMC8660796; doi:10.1038/s41467-021-27400-7)
Supplement: Supplementary file 3 — Reporting Summary [file 41467_2021_27400_MOESM3_ESM.pdf]

nature research

Corresponding author(s): Dr Chris H. Hill, Dr Stephen C. Graham, Prof Neva Caliskan, Prof Ian Brierley

Last updated by author(s): 20/10/2021

Reporting Summary

Nature Research wishes to improve the reproducibility of the work that we publish. This form provides structure for consistency and transparency in reporting. For further information on Nature Research policies, see our Editorial Policies and the Editorial Policy Checklist.

Statistics

For all statistical analyses, confirm that the following items are present in the figure legend, table legend, main text, or Methods section.

n/a	Confirmed	
☐	☒	The exact sample size (*n*) for each experimental group/condition, given as a discrete number and unit of measurement
☐	☒	A statement on whether measurements were taken from distinct samples or whether the same sample was measured repeatedly
☐	☒	The statistical test(s) used AND whether they are one- or two-sided<br>*Only common tests should be described solely by name; describe more complex techniques in the Methods section.*
☒	☐	A description of all covariates tested
☒	☐	A description of any assumptions or corrections, such as tests of normality and adjustment for multiple comparisons
☒	☐	A full description of the statistical parameters including central tendency (e.g. means) or other basic estimates (e.g. regression coefficient) AND variation (e.g. standard deviation) or associated estimates of uncertainty (e.g. confidence intervals)
☒	☐	For null hypothesis testing, the test statistic (e.g. *F*, *t*, *r*) with confidence intervals, effect sizes, degrees of freedom and *P* value noted<br>*Give P values as exact values whenever suitable.*
☒	☐	For Bayesian analysis, information on the choice of priors and Markov chain Monte Carlo settings
☒	☐	For hierarchical and complex designs, identification of the appropriate level for tests and full reporting of outcomes
☒	☐	Estimates of effect sizes (e.g. Cohen's *d*, Pearson's *r*), indicating how they were calculated

Our web collection on statistics for biologists contains articles on many of the points above.

Software and code

Policy information about availability of computer code

Data collection	X-ray crystallography: GDA (Diamond Light Source)<br>Electron Microscopy: EPU 2.0 (FEI)<br>SEC-MALS: ASTRA 6.1 (Wyatt Technologies)<br>Optical Tweezers: Bluelake (Lumicks)<br>MST: Monolith NT.115Pico control software (NanoTemper Technologies)<br>ITC: MicroCal PEAQ-ITC control software 1.29.32 (Malvern Panalytical)
Data analysis	SEC-MALS: ASTRA 6.1 (Wyatt Technologies)<br><br>X-ray crystallography: XIA2 (Winter, 2009), XDS (Kabsch, 2010), AIMLESS (Evans and Murshudov, 2013), autoSHARP (Vonrhein et al., 2007), SHELXD (Sheldrick, 2008), SHARP, SOLOMON (Abrahams and Leslie, 1996), ARP/wARP (Perrakis et al., 2001), Phenix 1.18.1_3865 [Phaser (McCoy et al., 2007), phenix.autobuild (Terwilliger et al., 2008), phenix.refine (Adams et al., 2010)]<br><br>Model building, validation and analysis: COOT 0.9.2 (Emsley et al., 2010), ISOLDE 1.1 (Croll et al., 2018), MolProbity (Chen et al., 2010), PDBeFOLD (Krissinel and Henrick, 2004), DALI (Holm and Laakso, 2016), PDBePISA (Krissinel and Henrick, 2007), CATHEDRAL (Redfern et al., 2007), PDB2PQR (Dolinsky et al., 2004), PROPKA (Li et al., 2005), APBS (Baker et al., 2001)<br><br>Cryo-EM: MotionCor 2 (Zheng et al., 2017), CtfFind4 (Rohou and Grigorieff, 2015), RELION 3.1 (Zivanov et al., 2018), Phenix 1.18.1_3865 (Adams et al., 2010)<br><br>Molecular Graphics: PyMOL 2.3.4 (Schrödinger LLC), ChimeraX 1.1 (Pettersen et al., 2004)<br><br>Gel electrophoresis band quantification: ImageQuantTM 8.1.0 (GE Healthcare), ImageStudio Lite 5.2 (LI-COR)

Plotting and curve-fitting and statistical analysis: Prism 8.0.2 (GraphPad), MO.Affinity Analysis (NanoTemper), MicroCal PEAQ-ITC analysis software 1.30 (Malvern Panalytical)

RNA structure prediction: RNAstructure 6.2 (https://rna.urmc.rochester.edu/RNAstructure.html), SimRNAweb (Magnus et al., 2016)

Optical Tweezers data: custom Python script, based on Pylake package provided by Lumicks (https://lumicks-pylake.readthedocs.io/). The force spectroscopy analysis scripts supporting the current study (Figure 3, Supplementary Figure 4, Supplementary Table 3) have been uploaded to GitHub [https://github.com/REMI-HIRI/EMCV_2A_project]. Further information is available on request from Neva Caliskan (neva.caliskan@helmholtz-hiri.de).

For manuscripts utilizing custom algorithms or software that are central to the research but not yet described in published literature, software must be made available to editors and reviewers. We strongly encourage code deposition in a community repository (e.g. GitHub). See the Nature Research guidelines for submitting code & software for further information.

Data

Policy information about availability of data

All manuscripts must include a data availability statement. This statement should provide the following information, where applicable:

- Accession codes, unique identifiers, or web links for publicly available datasets
- A list of figures that have associated raw data
- A description of any restrictions on data availability

Further information and requests for resources should be directed to and will be fulfilled by Ian Brierley (ib103@cam.ac.uk).

Materials availability:
Plasmids generated in this study (see methods), including those used to express recombinant 2A for purification, are available on reasonable request.
Unique reagents (anti-2A polyclonal antibody) are available on reasonable request. DNA and RNA oligonucleotides are standard synthetic products that are commercially available (see Supplementary Table 5).

Data availability:
Atomic coordinates and structure factors for the EMCV 2A X-ray crystal structure (Figure 1, Supplementary Figure 1, Supplementary Table 1) have been deposited in the wwPDB under accession code 7BNY [http://doi.org/10.2210/pdb7bny/pdb]. The 70S IC:2A cryo-EM map (Figure 5, Supplementary Figures 6, 7, Supplementary Table 4) has been deposited in the EMDB (EMD-12635, [https://www.ebi.ac.uk/emdb/EMD-12257]), and refined atomic coordinates accompanying this structure in the wwPDB (7NWT [http://doi.org/10.2210/pdb7nwt/pdb]). Previously-published structures that were used in this study are also available: 5WE6 [http://doi.org/10.2210/pdb5we6/pdb], 4V7D [http://doi.org/10.2210/pdb4v7d/pdb] and 5MDZ [http://doi.org/10.2210/pdb5mdz/pdb].

Raw optical tweezer data has been deposited in Mendeley Data [http://dx.doi.org/10.17632/gkpwngy65h.2] and is included in Source Data. Raw image data (e.g. uncropped, unannotated gels and western blots; Figures 1, 2, Supplementary Figures 2, 3 and 5) and MST traces (corresponding to individual panels in Figures 2 and 4) have been deposited in Mendeley Data [http://dx.doi.org/10.17632/gkpwngy65h.2] and are included in Source Data. See Additional Data statement at the end of the combined Supplementary Information File.

Field-specific reporting

Please select the one below that is the best fit for your research. If you are not sure, read the appropriate sections before making your selection.

☒ Life sciences ☐ Behavioural & social sciences ☐ Ecological, evolutionary & environmental sciences

For a reference copy of the document with all sections, see nature.com/documents/nr-reporting-summary-flat.pdf

Life sciences study design

All studies must disclose on these points even when the disclosure is negative.

Sample size	Molecular biology experiments were conducted in accordance with widely-accepted best practices [see e.g. Hill, C.H. et al. Mol Cell 73, 1217-1231 e11 (2019)]. No statistical methods were used to predetermine sample size. Sample sizes were chosen based on prior knowledge in the respective experiments and biological significance is only ascribed when the observed effects are large. For X-ray crystallography datasets, a 0.2 degree oscillation per image was used. Therefore, 900 images represent the required 180 degree oscillation range per dataset [see e.g. Hill, C.H. et al. Mol Cell 73, 1217-1231 e11 (2019)]. For cryo-EM, the reported 5730 micrographs collected is typical to achieve sufficient numbers of particles for high-resolution reconstruction [see e.g. Casanal, A. et al. Science 358, 1056-1059 (2017)].
Data exclusions	All cryo-EM micrographs were manually inspected. Micrographs were excluded from downstream analysis based on any of the following pre-established criteria:<br>i) physical damage (e.g. cracks) or contamination (e.g. ice, ethane) visible within the image<br>ii) evidence of crystalline/hexagonal ice in the FFT of the image<br>iii) unacceptable motion following MotionCor 2 analysis<br>iv) no visible particles<br>v) aggregated particles<br>vi) < 5A resolution by CTF estimation

April 2020

Replication	Unless otherwise stated in the text, all experiments were independently repeated at least two times. Image figures (e.g. gels, blots) are derived from a representative example a of replicated experiment.
Randomization	Not relevant as no experimental groups were used in this study. All experiments were conducted alongside negative and positive controls, as appropriate for each technique.
Blinding	The investigators were not blinded because collection and analysis of the presented data is not prone to bias or based on subjective assessment. Structural and molecular biology experiments are generally precise and quantitative. All were conducted alongside negative and positive controls, as appropriate for each technique.

Reporting for specific materials, systems and methods

We require information from authors about some types of materials, experimental systems and methods used in many studies. Here, indicate whether each material, system or method listed is relevant to your study. If you are not sure if a list item applies to your research, read the appropriate section before selecting a response.

Materials & experimental systems

n/a	Involved in the study	
☐	☒	Antibodies
☒	☐	Eukaryotic cell lines
☒	☐	Palaeontology and archaeology
☒	☐	Animals and other organisms
☒	☐	Human research participants
☒	☐	Clinical data
☒	☐	Dual use research of concern

Methods

n/a	Involved in the study	
☒	☐	ChIP-seq
☒	☐	Flow cytometry
☒	☐	MRI-based neuroimaging

Antibodies

Antibodies used

rabbit polyclonal anti-2A (Napthine et al., 2017) - custom product, raised against the C-terminal 14 aa of 2A by GenScript.
mouse monoclonal anti-RPS6, clone A16009C (BioLegend, 691802)
mouse monoclonal anti-RPL4, clone 4A3 (Sigma-Aldrich, WH0006124M1)
goat anti-rabbit IRDye 800 CW (LI-COR, 926-32211)
goat anti-mouse IRDye 680LT (LI-COR, 926-68020)

Validation

The anti-2A rabbit polyclonal antibody has been validated previously by Western-blot analysis of EMCV-infected BHK-21 cells (Napthine et al., 2017). In this current study, we confirmed this antibody recognises purified EMCV 2A following Western-blot analysis of 2A that had been recombinantly expressed in E.coli (relevant to Supplementary Figure 5).

The commercially-available mouse monoclonal anti-RPS6, clone A16009C (BioLegend, 691802, Mouse IgG2b, κ) has been raised against a Human ribosomal S6 peptide, and the manufacturer confirms Western blot cross-reactivity with human and mouse S6 protein (https://www.biolegend.com/en-us/products/purified-anti-rps6-antibody-13707). As QC, they demonstrate recognition of endogenous S6 protein in HeLa, Jurkat, NIH3T3 cells. They demonstrate a reduced intensity band following S6 knockdown in 293T cells. They provide the following relevant citation: Tsai S, et al. 2018. Cell Metab. 28:922
We demonstrate here that it also cross-reacts with rabbit S6 protein by Western-blot analysis of 40S ribosomal subunits purified from rabbit reticulocyte lysate (Supplementary Figure 5).

The commercially-available mouse monoclonal anti-RPL4, clone 4A3 (Sigma-Aldrich, WH0006124M1, IgG2aκ) has been raised against a recombinant fragment of RPL4 (NP_000959, 251 a.a. - 350 a.a) with a GST tag, and the manufacturer confirms Western blot cross-reactivity against human, mouse and rat L4 protein (https://www.sigmaaldrich.com/catalog/product/sigma/wh0006124m1).
We demonstrate here that it also cross-reacts with rabbit L4 protein by Western-blot analysis of 60S ribosomal subunits purified from rabbit reticulocyte lysate (Supplementary Figure 5).
