# Supplementary material for: Structural and molecular basis for Cardiovirus 2A protein as a viral gene expression switch
Source: Nat Commun. 2021 Dec 9;12:7166. doi: 10.1038/s41467-021-27400-7 (PMC8660796; doi:10.1038/s41467-021-27400-7)
Supplement: Supplementary file 4 — Source Data [file 41467_2021_27400_MOESM4_ESM.zip › Source_data/Figure2.zip!/Figure2/Raw MST traces and capillary scans.pdf]

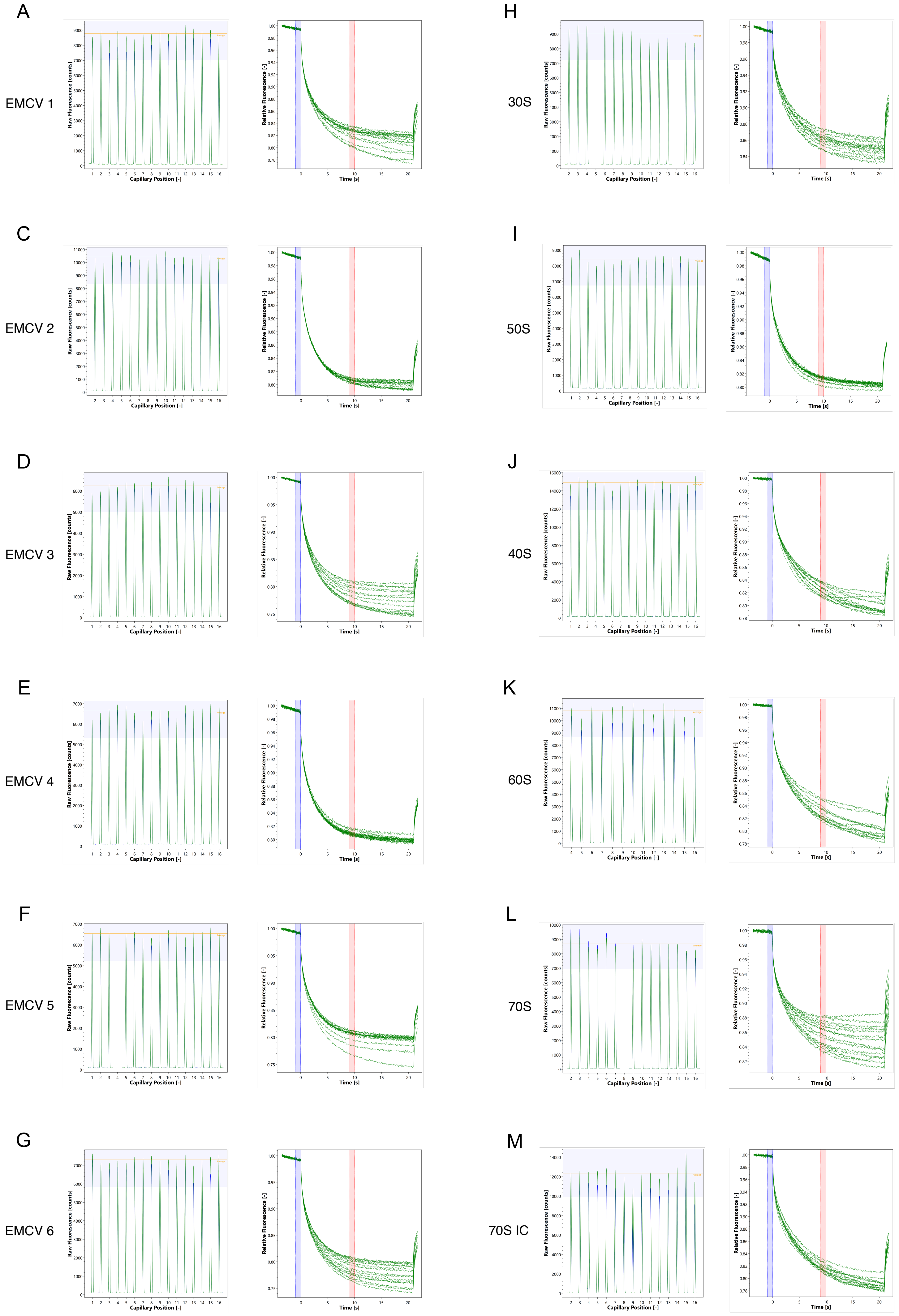
A
EMCV 1
C
EMCV 2
D
EMCV 3
E
EMCV 4
F
EMCV 5
G
EMCV 6
H
30S
I
50S
J
40S
K
60S
L
70S
M
70S IC
Raw Fluorescence [counts]
Capillary Position [-]
Relative Fluorescence [-]
Time [s]
